# Enhancing performance and uniformity of CH_3_NH_3_PbI_3−x_Cl_x_ perovskite solar cells by air-heated-oven assisted annealing under various humidities

**DOI:** 10.1038/srep21257

**Published:** 2016-02-16

**Authors:** Qing Zhou, Zhiwen Jin, Hui Li, Jizheng Wang

**Affiliations:** 1 Beijing National Laboratory for Molecular Sciences, CAS Key Laboratory of Organic Solids, Institute of Chemistry, Chinese Academy of Sciences, Beijing 100190, China; 2 University of Chinese Academy of Sciences, Beijing 100049, China

## Abstract

To fabricate high-performance metal-halide perovskite solar cells, a thermal annealing process is indispensable in preparing high quality perovskite film. And usually such annealing is performed on hot plate. However hot-plate annealing could cause problems such as inhomogeneous heating (induced by non-tight contact between the sample and the plate), it is also not fit for large scale manufactory. In this paper, we conduct the annealing process in air-heated oven under various humidity environments, and compared the resulted films (CH_3_NH_3_PbI_3−x_Cl_x_) and devices (Al/PC_61_BM/CH_3_NH_3_PbI_3−x_Cl_x_/PEDOT:PSS/ITO/glass) with that obtained via hot-plate annealing. It is found that the air-heated-oven annealing is superior to the hot-plate annealing: the annealing time is shorter, the films are more uniform, and the devices exhibit higher power conversion efficiency and better uniformity. The highest efficiencies achieved for the oven and hot-plate annealing processes are 14.9% and 13.5%, and the corresponding standard deviations are 0.5% and 0.8%, respectively. Our work here indicates that air-heated-oven annealing could be a more reliable and more efficient way for both lab research and large-scale production.

Nowadays many scientists and engineers worldwide are working on development of photovoltaic devices, which are promising in providing clean energy for human beings in the future. Metal-halide perovskite solar cells have recently emerged at the forefront of such photovoltaic research, and in a very short time of about 5 years, their power conversion efficiencies have skyrocketed from about 3% to higher than 20%^1^,^2^,^3^,^4^,^5^,^6^,^7^. To fabricate high-performance perovskite solar cells, the most crucial step is to form high quality crystallinity perovskite film (dense, pure, uniform), for which a thermal annealing process is indispensable no matter how the initial film is formed (vapor-deposited process^8^,^9^,^10^, one-step solution process^11^,^12^,^13^,^14^, two-step depositing^3^,^15^,^16^, solution-engineering^5^,^6^,^17^, printing approach^18^,^19^,^20^,^21^,^22^ and solution-annealing postprocessing^23^,^24^). Usually the annealing is done on hot plate. However, annealing on hot plate is not suitable for large scale production and could have drawbacks that are difficult to overcome such as inhomogeneous heating induced by non-tight contact between the sample and the plate. Doing the annealing in air-heated oven could be a better approach. However, it has been seldom employed^25^,^26^, not to mention detailed investigation.

Here we do a brief analysis and comparison between the two approaches. In case of hot-plate annealing, the bottom of the sample directly contacts the surface of the plate, and the other side of the sample directly exposed to air (or nitrogen gas), the air nearby the surface could be heated hot, but the air temperature should gradually decrease away from the sample surface. Hence it is reasonable to state that the temperature must have certain gradients from the very bottom of the sample to the surface of the sample to the air environment. So the whole system (sample and the air environment) is not uniform in temperature. Whereas in case of air-heated-oven annealing, hot air with well-controlled temperature is filled into the oven and heated everything inside the oven, and hence the whole system is uniform in temperature. Also for the hot-plate annealing, the sample must closely contact the surface of the hot plate, which requires very flat surfaces for both the hot plate and the bottom of the sample. This sometimes is quite difficult to satisfy since small particles and other contaminations all can damage the flatness of these surfaces. Hence uniform heating for even one sample could somehow be hard to guarantee, not to mention uniform heating for different samples annealed at different batches. Such problem could seriously damage the performance uniformity of the annealed films and their corresponding devices. And in most cases one cannot put many samples on a hot plate at one time. These problems can all be avoided in air-heated oven. The samples can be put in any positions and in any postures in the oven, making the annealing process much more convenient and controllable, which is very suitable for large-scale production.

In this study, PbCl_2_ + 3CH_3_NH_3_I precursor films on PEDOT:PSS/ITO/glass substrates were annealed both on hot plate and in air-heated oven under various humidity (It is reported that suitable humidity during the film annealing can benefit the device performance due to formation of better-morphology films^27^,^28^,^29^,^30^,^31^,^32^,^33^,^34^). The annealing process is to release the redundant organic halide CH_3_NH_3_Cl or other unknown species^35^,^36^ and finally form the CH_3_NH_3_PbI_3−*x*_Cl_*x*_ perovskite films (seen in Fig. 1). We found that the oven annealing results in more uniform CH_3_NH_3_PbI_3−*x*_Cl_*x*_ films with fewer pin-holes, the corresponding perovskite solar cells also exhibit superior performances in efficiency and uniformity. Meanwhile, we systematically investigated the humidity effects on the film morphology and the device performance.

## Results and Discussion

**Figure 2:**
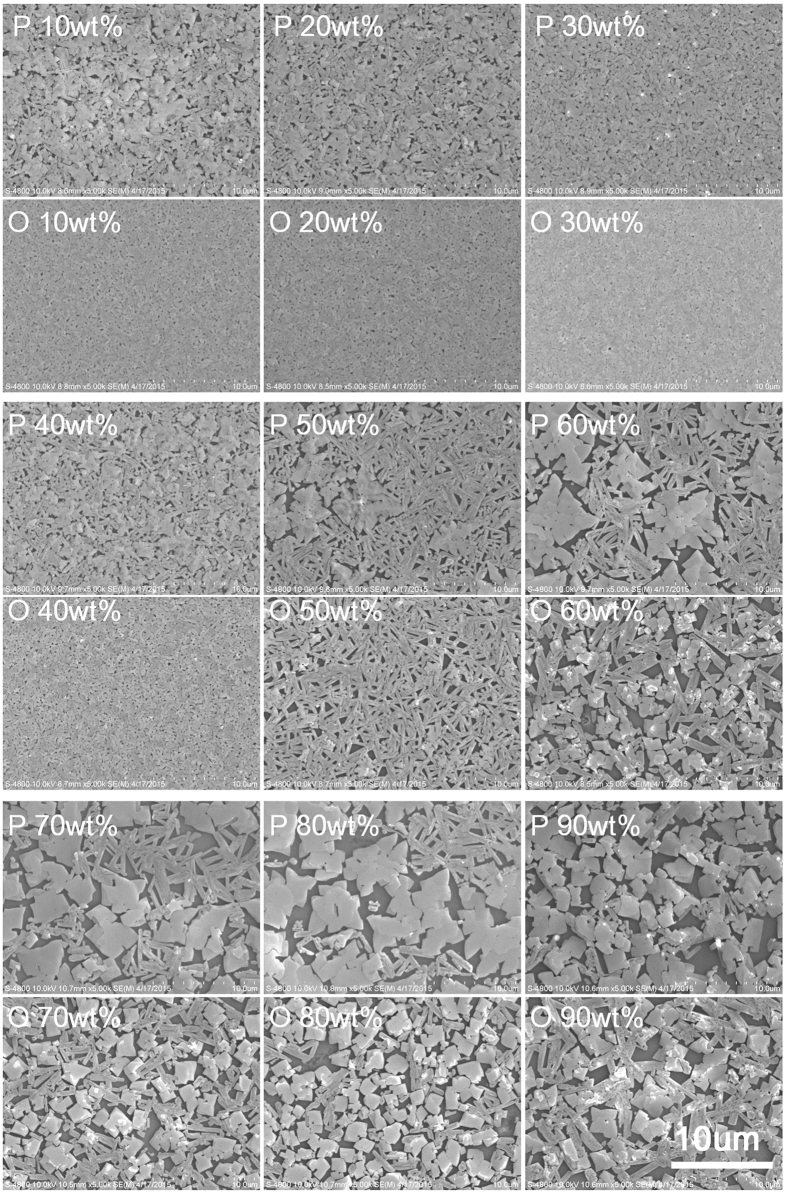
SEM images of the CH_3_NH_3_PbI_3−x_Cl_x_ perovskite films annealed under different humidity at 100 °C on hot plate for 35 min (upper row of each group) and in air-heated oven for 25 min (lower row of each group). The magnification for all the images is the same and the scale bar is shown in the last image.

Figure 2 gives the Scanning Electron Microscopy (SEM) of the P films (films annealed on the hot plate) and the O films (films annealed in the oven) in different humidity environments. It is seen that for the P films, there are many small pin-holes under a relative humidity (RH) of 10%, and there are less and less pin-holes with the RH increasing to 20% and then 30%, and their sizes becomes smaller and smaller. The tide reverses when the RH is further increased to 40%: more pin-holes appear and their sizes increase. Meanwhile, relative large crystals start to form and the film seemingly loses its continuity. The tendency continues with increasing the RH to 50%, larger crystals form and the film breaks in certain areas. At 60% RH, it can be clearly seen that the film actually really breaks in most areas, and even larger crystals form with well-discerned gaps between them. With further increasing the humidity (70% to 90% RH), larger and larger crystals form with bigger and bigger gaps between them, and the film completely breaks in all the areas. For the O films, the overall tendency of the film evolution with humidity is the same. In comparison, the O film is more uniform than the P film for all the humidity. Under 10% to 40% RH, the O films are all very uniform with much fewer and smaller pin-holes, especially for the case of 30% RH, the film is very uniform and almost pin-hole free. Even at 40% RH (under which the P film already starts to break), the film is clearly continuous with only very small pin-holes. Even for the crystals formed at high humidity levels (50% to 90% RH), they are much more uniform in size for the O films. From these images it can also be concluded that the humidity has great influence over the formation of the perovskite film during the annealing process. And it is clear that for both the O films and the P films, a RH of 30% is optimal.

**Figure 3:**
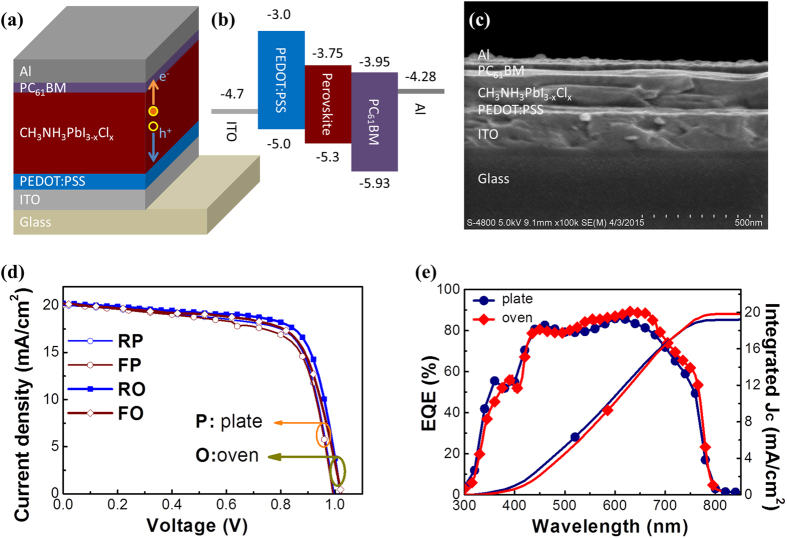
(**a**) Device structure of the perovskite inverted planar devices. (**b**) Energy level diagram of the device. (**c**) Cross-sectional SEM image of the device. (**d**) *J-V* curves of the best devices tested under 1.5 AM at 100 mW/cm^2^ illumination (scan rate: 200 mV/s; RP/RO: reverse scan of the P/O device; FP/FO: forward scan of the P/O devices). (**e**) EQE of the best devices. The integrated *J*_*SC*_ are 19.5 mA/cm^2^ and 19.2 mA/cm^2^ for the O device and the P device, respectively.

Photovoltaic devices were then fabricated based on the P films and the O films. The devices were structured as Al/PC_61_BM/CH_3_NH_3_PbI_3−x_Cl_x_/PEDOT:PSS/ITO/glass (shown in Fig. 3a). The energy level diagram of the device is displayed in Fig. 3b. The cross sectional SEM image of one O device (with the film formed at 30% RH) is given in Fig. 3c, in which each layer can be clearly discerned. And the thickness of CH_3_NH_3_PbI_3−x_Cl_x_ film is about 260 nm. We found for both the O devices and the P devices, the best performance was observed for the film formed under 30% RH. *J-V* curves of the two best devices are given in Fig. 3d. The P device shows a PCE of 13.5%: *V*_*OC*_ =  0.98 V, *J*_*SC*_ = 20.1 mA/cm^2^, and FF = 0.69. The O device exhibits a PCE of 14.9%: *V*_*OC*_ = 1.02 V, *J*_*SC*_ = 20.3 mA/cm^2^, FF = 0.72. It is seen that *V*_*OC*_, *J*_*SC*_ and FF are all improved by air-heated-oven annealing, and as a result an over 10% improvement in PCE is achieved. EQE of the two best devices are presented in Fig. 3e, together with the integrated current density as a function of wavelength^37^. The integrated *J*_*SC*_ is 19.5 and 19.2 mA/cm^2^ for the O device and the P device, respectively. They are both slightly smaller than the measured ones from the *J-V* curves. This could be caused by device degradation during their exposure in air (during the transfer and EQE test process). The EQE spectrum show the onset of photocurrent at 790 nm, consistent with the reported band gap of CH_3_NH_3_PbI_3−x_Cl_x_^25^.

**Figure 4:**
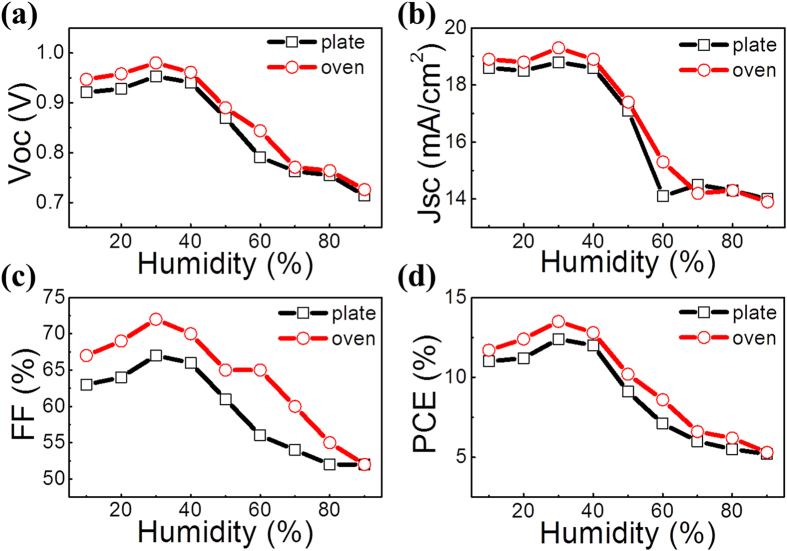
Statistics (**a**) *V*_*OC*_, (**b**) *J*_*SC*_, (**c**) FF, and (**d**) PCE dependence on the humidity. In the study, 40 devices are used for both the O and the P devices under each humidity condition.

To investigate uniformity of the device performance, 40 O devices/40 P devices from 3 batches are used to do the statistic study for each humidity condition. Average values of the device parameters (including *J*_*SC*_, *V*_*OC*_, FF and PCE) are summarized in Table 1, and are plotted in Fig. 4 as circles and squares. It is seen that overall for both the O devices and the P devices, the average *V*_*OC*_, *J*_*SC*_, FF and PCE all increase with the humidity in the range of 10% to 30% RH, and then drop with further increasing the humidity (30% to 90% RH). In comparison, the O device almost always shows superior *V*_*OC*_/*J*_*SC*_/FF/PCE over the P device under each humidity condition. Backing to Fig. 2, it can be easily found: 1) the dependence of the performance and its uniformity on the humidity is basically consistent with uniformity of the film morphology: the more uniform the film, the better the device performance as well as the performance uniformity. 2) The dependence of the performance and its uniformity on the annealing approach is also fundamentally consistent with uniformity of the film morphology: the more uniform the film, the better the device performance as well as the performance uniformity. Surprisingly, at very high humidity of 90% RH, the device still shows a PCE of around 5%. As can be imaged, the large gap area between the crystals might be covered by a CH_3_NH_3_PbI_3−x_Cl_x_ thin film, which together with the PC_61_BM layer could prevent possible large current leakage between the anode and the cathode, and hence the device still can maintain a normal behavior. However, light current generated in the gap area should be very small and dark current in these areas should be quite large. Thereby these gap areas play a negative role of dragging down the overall light current density of the device and pushing up the overall dark current density of the device, and as a result, leading to smaller *J*_*SC*_, *V*_*OC*_, and FF.

**Figure 5:**
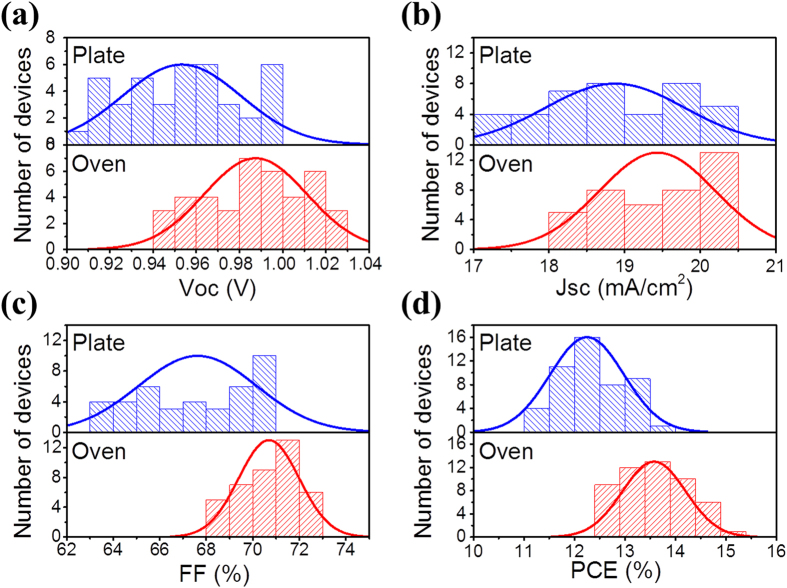
Histograms of device parameters measured for 40 O and 40 P devices in 30% RH. (**a**) *V*_*OC*_, (**b**) *J*_*SC*_, (**c**) FF and (**d**) PCE. The Gaussian fits are presented as a guide to the eye.

In Table 1, the standard deviation values of the device parameters were also provided. It is seen that for all the humidity, the O devices exhibit better uniformity indicated by the smaller standard deviations of all the parameters (*J*_*SC*_, *V*_*OC*_, FF and hence PCE). As a representative, histograms of the statistic are shown in Fig. 5 for the O devices and the P devices under relative humidity 30%. As can be clearly seen, the O devices have much better performance and much better uniformity for all the parameters. For example, the PCE varies in a range of 12.4% to 14.9% with a deviation of 0.5%, whereas that of the P device is 11.3% to 13.5% with a deviation of 0.8%. The FF shows even more remarkable difference: 68% to 73% with a deviation of ~1.3% vs 63% to 71% with a deviation of 2.6%. Here we list the minimum and maximum values of the device parameters and their corresponding standard deviation Δ for the O/P devices. For the O devices, *V*_*OC*_: 0.945 − 1.022 V, Δ = 0.024 V; *J*_*SC*_: 18.1 − 20.5 mA/cm^2^, Δ = 0.8 mA/cm^2^; and FF: 0.68 − 0.73, Δ = 0.01; PCE: 12.5% – 14.9%, Δ = 0.6%; for the P devices, *V*_*OC*_: 0.901 – 1.000 V, Δ = 0.03 V; *J*_*SC*_: 17.4 – 20.3 mA/cm^2^, Δ = 0.9 mA/cm^2^; FF: 0.63 – 0.71, Δ = 0.03; PCE: 10.4% – 13.5%, Δ = 0.8%.

## Conclusions

In summary, air-heated-oven annealing is employed in preparing CH_3_NH_3_PbI_3−x_Cl_x_ films for high-performance perovskite solar cells (Al/PC_61_BM/CH_3_NH_3_PbI_3−x_Cl_x_/PEDOT:PSS/ITO/glass). The resulted CH_3_NH_3_PbI_3−x_Cl_x_ films under a wide range of humidity (10% to 90% RH) are more uniform than the corresponding ones obtained via the popular hot-plate annealing. Accordingly, the fabricated devices display better performance than the corresponding ones fabricated via hot-plate annealing: higher PCE and higher uniformity. A high PCE of 14.9% is achieved (*V*_*OC*_ of 1.02 V, *J*_*SC*_ of 20.3 mA/cm^2^, FF of 0.72), which is amongst the highest for such device with the structure of Al/PC_61_BM/CH_3_NH_3_PbI_3−x_Cl_x_/PEDOT:PSS/ITO/glass. In contrast, the best device fabricated via hot-plate-annealing shows an inferior PCE of 13.5% (*V*_*OC*_ of 0.98 V, *J*_*SC*_ of 20.1 mA/cm^2^, FF of 0.69). We also systematically investigated the humidity effects on film morphology and device performance. The work demonstrates that air-heated-oven annealing we employed here could have great applications in future perovskite solar cell fabrications.

## Experimental

### CH_3_NH_3_I synthesis

Methylammonium iodide (MAI) was synthesized by reacting 10 mL hydroiodic acid (57% in water, Sigma-Aldrich), 24 mL methylamine (33% in absolute ethanol, Sigma-Aldrich), and 100 mL ethanol in a 250 mL round-bottomed flask at 0 °C for 2 h with stirring. The white precipitate of MAI was obtained by rotary evaporating the solution at 50 °C for about 1 h. The product was dissolved in ethanol, recrystallized by sedimentation in diethyl ether, and dried at 60 °C under vacuum for 24 h.

### Precursor solution preparing

MAI and lead (II) chloride (PbCl_2_, Sigma-Aldrich, 99.9995%) were dissolved in anhydrous N,N-Dimethylformamide (DMF, Aldrich), with a molar ratio of 3:1 and a final weight percent of 30% in DMF. This solution was filtered through a 0.4-μm-pore PTFE filter after stirring at 60 °C for 24 h and stored under a dry nitrogen atmosphere.

### Film depositing and annealing

The patterned ITO (15 ohm/sq) glass substrates were washed sequentially with detergent and deionized water, acetone, and isopropanol with ultrasonication for 10 min each, and then were dried and treated by O_2_ plasma. A dispersion of poly(3,4-ethylenedioxythiophene):poly(styrenesulfonic acid) (PEDOT:PSS, levios, Al 4083, filtered through a 0.45 μm nylon filter) was first spin-coated on the ITO substrate at 2000 rpm for 45 s and subsequently dried at 140 °C for 15 min in air. To avoid the effect of moisture on perovskite precursor solution during the fabrication of perovskite thin-film, the substrates were transferred into a N_2_ glove-box, where the perovskite film (about 260 nm thickness) was fabricated by spin-coating a 30 wt% CH_3_NH_3_PbI_3−x_Cl_x_ precursor solution (PbCl_2_ + 3CH_3_NH_3_I) at 3000 rpm for 45 s. The samples were then transferred out of the N_2_ glove-box and put in a dry-air supplied glove-box, then were annealed in an air-heated oven (with forced air circulation, LABCAB, DHG9030A) (at 100 °C for 25 min) or on a hot plate (Stuart SD160, UK) (at 100 °C for 35 min) under various humidity condition (10% to 90% RH). The optimization of annealing process and characterizations of corresponding materials and films can be found in the supporting information Figure S1–S4.

### Device fabrication

After annealing, the [6,6]-phenyl-C61-butyric acid methyl ester (PC_61_BM, 15 mg/mL in chlorobenzene) solution was spin-coated on top of the perovskite layer at 900 rpm for 18 s and 6000 rpm for 30 s sequentially in the N_2_ glove-box. Finally, 100 nm thick aluminum cathode electrode was deposited by thermal evaporation through a metal shadow mask, completing the fabrication of the device (Al/PC_61_BM/CH_3_NH_3_PbI_3−x_Cl_x_/PEDOT:PSS/ITO/ glass).

### Relative Humidity Control

For all the humidity studies, the perovskite films were annealed in a 1.8 × 0.8 × 0.6 m^3^ controlled-humidity (measured at 26 ± 1 °C) air glove-box. The relative humidity was carefully adjusted and controlled by evaporating deionized water in dry-air supplied glove-box, and waiting for more than 30 min to reach equilibrium. The relative humidity was measured periodically using a hygrometer throughout the course of the experiments using a calibrated hygrometer (±2%).

### Material and film Characterization

The X-ray diffraction (XRD) spectra of the PbI_2_ powder and the prepared films were measured using Rigaku-2500 X-ray diffractometer with an X-ray tube (Cu Kα, λ = 1.5406 Å). The morphology of the films was recorded using a scanning electron microscope (SEM, HITACH2100). X-ray photoelectron spectroscopy (XPS) was carried out in ESCALab250Xi XPS system by a MgKα X-ray source (1253.6 eV).

### Device Characterization

External quantum efficiency (EQE) was recorded by a Newport Oriel IQE-200 by a power source (Newport 300 W Xenon lamp, 66920) with a monochromatic (Newport Cornerstone 260). All current density-voltage (*J-V)* curves were measured using a source meter (Keithley 2420, USA) under AM 1.5 sunlight at an irradiance of 100 mW/cm^2^ provided by a solar simulator (Newport, Oriel Sol3A Class AAA, 94043A). Light intensity was calibrated using a monocrystalline silicon reference cell with KG5 window (Newport, Oriel 91150). The *J-V* curves were measured by reverse (forward bias (1.2 V) → backward voltage (−0.8 V)) or forward (backward voltage (−0.8 V) → forward bias (1.2 V)) scan (with a rate of 200 mV/s). The device area was 0.168 cm^2^, determined by the overlap of the cathode and anode. In order to avoid the overestimation of the photocurrent density by the edge effect and the optical piping effect, the solar cells were masked with a metal aperture (area of 0.08 cm^2^) over the device (0.168 cm^2^) to define the active area^38^,^39^.

### Device parameters statistics

Mean (average) values and standard deviations of *V*_*OC*_/*J*_*SC*_/FF/PCE for the CH_3_NH_3_PbI_3−x_Cl_x_ perovskite solar cell based on both annealing approaches. Data were statistically analyzed with outlying observation using the descriptive statistics in Origin Version 9.0. Standard deviation is a widely used measure of variability and uniformity used in statistics theory. The smaller the standard deviation, the more uniform the parameter is.

## Additional Information

**How to cite this article**: Zhou, Q. *et al.* Enhancing performance and uniformity of CH_3_NH_3_PbI_3−x_Cl_x_ perovskite solar cells by air-heated-oven assisted annealing under various humidities. *Sci. Rep.* **6**, 21257; doi: 10.1038/srep21257 (2016).

## Supplementary Material

Supplementary Information

## Figures and Tables

**Figure 1:**
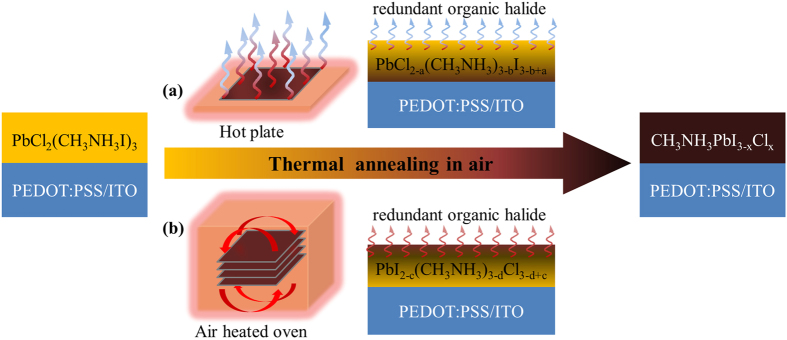
Schematic of the annealing approaches. (**a**) hot plate. (**b**) air-heated oven.

**Table 1:** Statistics from 40 O/40 P devices for each humidity: average values of *V*_*OC*_ *, J*_*SC*_, FF and PCE and the standard deviations (average value ± standard deviation).

	Humidity (%)	*V*_*OC*_ (V)	*J*_*SC*_ (mA/cm^2^)	FF (%)	PCE (%)	PCE (%) best
hot plate	10	0.921 ± 0.035	18.6 ± 0.9	63 ± 4	11.0 ± 0.9	12.2
	20	0.928 ± 0.034	18.5 ± 0.9	64 ± 4	11.2 ± 0.9	13.1
	30	0.954 ± 0.028	18.9 ± 0.9	67 ± 3	12.2 ± 0.8	13.5
	40	0.941 ± 0.030	18.6 ± 1.0	66 ± 3	12.0 ± 0.9	13.1
	50	0.870 ± 0.040	17.1 ± 1.0	61 ± 5	9.1 ± 1.0	11.7
	60	0.791 ± 0.067	14.1 ± 1.2	56±6	7.1 ± 1.1	9.2
	70	0.763 ± 0.082	14.5 ± 1.3	54 ± 6	6.0 ± 1.1	9.0
	80	0.755 ± 0.088	14.3 ± 1.5	52 ± 6	5.5 ± 1.0	7.9
	90	0.714 ± 0.088	14.0 ± 1.6	52 ± 6	5.2 ± 1.1	7.2
air-heated oven	10	0.947 ± 0.032	18.9 ± 0.6	67 ± 4	11.7±0.6	12.7
	20	0.958 ± 0.027	18.8 ± 0.8	69 ± 3	12.4 ± 0.6	13.8
	30	0.987 ± 0.024	19.3 ± 0.8	71±1	13.6 ± 0.6	14.9
	40	0.961 ± 0.025	18.9 ± 0.8	70 ± 2	12.7 ± 0.7	13.7
	50	0.890 ± 0.031	17.4 ± 0.8	65 ± 3	10.2 ± 0.7	12.1
	60	0.884 ± 0.039	15.3 ± 0.9	65 ± 4	8.6 ± 0.8	11.6
	70	0.771 ± 0.043	14.2 ± 1.1	60 ± 4	6.6 ± 0.8	9.2
	80	0.764 ± 0.047	14.3 ± 1.0	55 ± 5	6.2 ± 0.8	8.3
	90	0.726 ± 0.047	13.9 ± 1.4	52 ± 6	5.3 ± 0.8	7.6
