# Supplementary material for: Enhancing performance and uniformity of CH3NH3PbI3−xClx perovskite solar cells by air-heated-oven assisted annealing under various humidities
Source: Sci Rep. 2016 Feb 16;6:21257. doi: 10.1038/srep21257 (PMC4754658; doi:10.1038/srep21257)
Supplement: Supplementary Information [file srep21257-s1.pdf]

Supplementary Information

Enhancing performance and uniformity of $CH_3NH_3PbI_{3-x}Cl_x$ perovskite solar cells by air-heated-oven assisted annealing

Qing Zhou[1,2], Zhiwen Jin[1,2], Hui Li[1] and Jizheng Wang[1]*

[1]Beijing National Laboratory for Molecular Sciences, CAS Key Laboratory of Organic Solids, Institute of Chemistry, Chinese Academy of Sciences, Beijing 100190, China.

[2]University of Chinese Academy of Sciences, Beijing 100049, China.

Email: jizheng@iccas.ac.cn

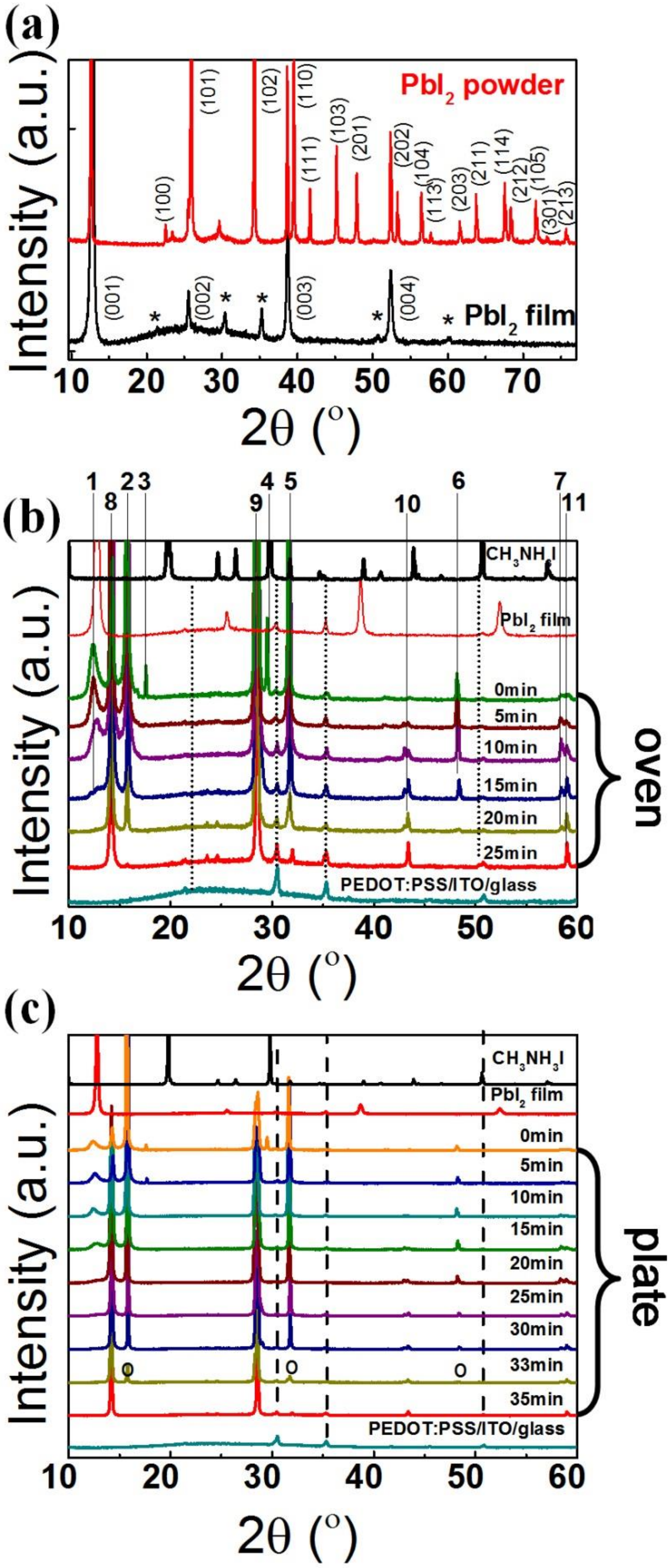

Figure S1 XRD spectrum. (a) PbI_2 power and PbI_2 film. (b) air-heated-oven annealed precursor films. (c) hot-plate annealed precursor films.

We performed XRD measurements for the PbI_2 powder and compared the spectrum with that of the PbI_2 film obtained by solution spin-coating process (**Figure S1a**): less peaks are observed for the film due to the fact that in the film, most crystal facing up as (001), thereby signals from other faces will be greatly weakened or diminished. This is also the reason that PbI_2 single crystal can be fabricated by simple spin-coating process.[1, 2] Note that both the PbI_2 powder and the film look yellowish. [3] It is found that structure of the PbI_2 crystal grown by this craft is 2H with hexagonal space group P-3m1, and the lattice constants were calculated to be a=0.4557nm and c=0.6980nm, in agreement with the PDF values. Usually, PbI_2 crystal presents three polytypes of 2H, 4H and 12R, in which 2H is the stable structure of the crystal at room temperature.[4, 5]According to literature data, the PbI_2 film deposited by spin-coating from DMF solution crystallizes in the form of the hexagonal 2H polytype, the most common PbI_2 modification (Inorganic Crystal Structure Database, collection code 68819). [6] The results show that on a flat substrate, crystals grow in a preferential orientation along the *c*-axis, hence only four diffraction peaks appeared, corresponding to the (001), (002), (003) and (004) lattice planes, the cleavage plane of PbI_2 single crystal [4]

Figure S1b shows the X-ray diffraction of the O films annealed at 100 ℃ with different annealing time, in which that of the PEDOT:PSS/ITO/glass substrate, the CH_3NH_3I film and the PbI_2 film are also given. It is seen that the XRD of the original film actually does not contain any signals from CH_3NH_3I and PbI_2, and no XRD signals of $CH_3NH_3PbI_{3-x}Cl_x$ are observed neither. This indicates that during

spin-coating the CH_3NH_3I / PbI_2 mixed solution, the two materials already react with each other and some new materials that is different with $CH_3NH_3PbI_{3-x}Cl_x$ is produced, which have several XRD peaks located at, marked as 1, 2, 3, 4, 5, 6, 7 respectively. Generally, it is concluded that the spin-coated CH_3NH_3I / PbI_2 (ratio of 3:1 in DMF) film contains CH_3NH_3I and PbI_2, during annealing the two materials starts to react, and $CH_3NH_3PbI_{3-x}Cl_x$ is generated. The extra CH_3NH_3I is released into the environment. In this paper, we are not concentrating on the new materials; however we wish our findings here would be interesting to the community. With increasing the annealing time (5-25 min), peak 1 to 7 vanished gradually one by one, and signals of $CH_3NH_3PbI_{3-x}Cl_x$ (peak 8, 9, 10, 11) appears and become more and more dominant, and finally only signals of $CH_3NH_3PbI_{3-x}Cl_x$ (except peaks from the PEDOT:PSS/ITO/glass) are observed in the spectra. This indicates that the reaction is complete and pure $CH_3NH_3PbI_{3-x}Cl_x$ film is formed. For the P films, the same tendency is observed, only the corresponding annealing times are different (see **Figure S1c**). The optimal annealing time to form pure $CH_3NH_3PbI_{3-x}Cl_x$ film is 35 min, longer than that of air-heated-oven annealing.

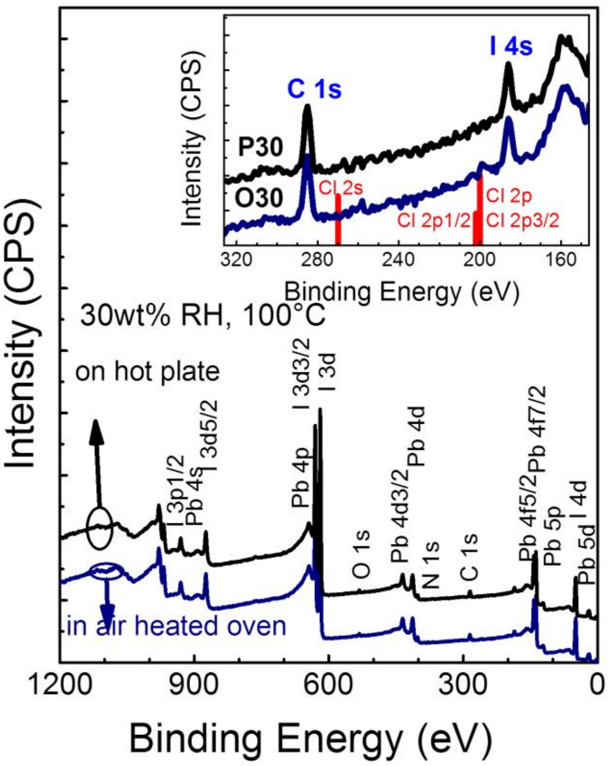

Figure S2 X-ray Photoelectric Spectroscopy (XPS) of the O film (blue) and the P film (black).

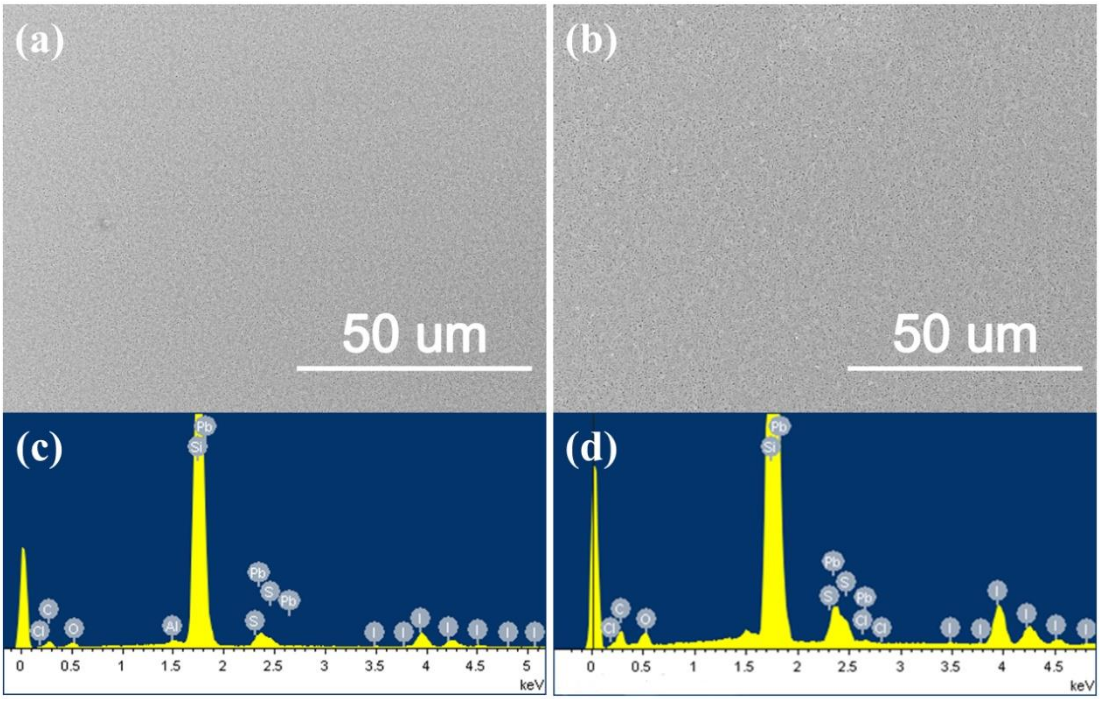

Figure S3 Energy-dispersive x-ray spectroscopy (EDS) with confocal fluorescence maps of the O film (a)/(c) and the P film (b)/(d).

Figure S2 presents XPS of the P film and the O film. It is seen that both films do not contain observable signals from element Cl (the position of the Cl XPS signals are marked as three vertical red lines), indicating the nominal $CH_3NH_3PbI_{3-x}Cl_x$ mostly should be $CH_3NH_3PbI_3$, or x is negligible. This is consistent with the reported.[7] We have used energy-dispersive X-ray spectroscopy (EDS) to confirm the above results (**Figure S3**), in which signals from Cl is negligible. So $PbCl_2$ actually plays a role of providing Pb, analogous to the role of lead acetate [$Pb(Ac)_2$] reported elsewhere.[8-10]

Reference

1. Nie, W. *et al.* High-efficiency solution-processed perovskite solar cells with millimeter-scale grains. *Science* **347**, 522-525, (2015).
2. Conings, B. *et al.* Perovskite-Based Hybrid Solar Cells Exceeding 10% Efficiency with High Reproducibility Using a Thin Film Sandwich Approach. *Adv. Mater.* **26**, 2041-2046, (2013).
3. Condeles, J. F., Lofrano, R. C. Z., Rosolen, J. M. & Mulato, M. Stoichiometry, surface and structural characterization of lead iodide thin films. *Braz. J. Phys.* **36**, 320-323, (2006).
4. Zhu, X. H., Wei, Z. R., Jin, Y. R. & Xiang, A. P. Growth and characterization of a PbI2 single crystal used for gamma ray detectors. *Cryst. Res. Technol.* **42**, 456-459, (2007).
5. Beckmann, P. A. A review of polytypism in lead iodide. *Cryst. Res. Technol.* **45**, 455-460, (2010).
6. Burschka, J. *et al.* Sequential deposition as a route to high-performance perovskite-sensitized solar cells. *Nature* **499**, 316-319, (2013).
7. Wang, B., Young Wong, K., Xiao, X. & Chen, T. Elucidating the Reaction Pathways in the Synthesis of Organolead Trihalide Perovskite for High-Performance Solar Cells. *Sci. Rep.* **5**, 10557, (2015).
8. Buin, A. *et al.* Materials Processing Routes to Trap-Free Halide Perovskites. *Nano Lett.* **14**, 6281-6286, (2014).
9. Zhang, W. *et al.* Ultrasmooth organic-inorganic perovskite thin-film formation and crystallization for efficient planar heterojunction solar cells. *Nat Commun* **6**, 6142, (2015).
10. Aldibaja, F. K. *et al.* Effect of different lead precursors on perovskite solar cell performance and stability. *J. Mater. Chem. A* **3**, 9194-9200, (2015).
